# The Effects of the Biceps Brachii and Brachioradialis on Elbow Flexor Muscle Strength and Spasticity in Stroke Patients

**DOI:** 10.1155/2022/1295908

**Published:** 2022-03-02

**Authors:** Binbin Yu, Xintong Zhang, Yihui Cheng, Lingling Liu, YanJiang, Jiayue Wang, Xiao Lu

**Affiliations:** Department of Rehabilitation Medicine, The First Affiliated Hospital of Nanjing Medical University, Jiangsu, China

## Abstract

**Objective:**

Muscle weakness and spasticity are common consequences of stroke, leading to a decrease in physical activity. The effective implementation of precision rehabilitation requires detailed rehabilitation evaluation. We aimed to analyze the surface electromyography (sEMG) signal features of elbow flexor muscle (biceps brachii and brachioradialis) spasticity in maximum voluntary isometric contraction (MVIC) and fast passive extension (FPE) in stroke patients and to explore the main muscle groups that affect the active movement and spasticity of the elbow flexor muscles to provide an objective reference for optimizing stroke rehabilitation.

**Methods:**

Fifteen patients with elbow flexor spasticity after stroke were enrolled in this study. sEMG signals of the paretic and nonparetic elbow flexor muscles (biceps and brachioradialis) were detected during MVIC and FPE, and root mean square (RMS) values were calculated. The RMS values (mean and peak) of the biceps and brachioradialis were compared between the paretic and nonparetic sides. Additionally, the correlation between the manual muscle test (MMT) score and the RMS values (mean and peak) of the paretic elbow flexors during MVIC was analyzed, and the correlation between the modified Ashworth scale (MAS) score and the RMS values (mean and peak) of the paretic elbow flexors during FPE was analyzed.

**Results:**

During MVIC exercise, the RMS values (mean and peak) of the biceps and brachioradialis on the paretic side were significantly lower than those on the nonparetic side (*p* < 0.01), and the RMS values (mean and peak) of the bilateral biceps were significantly higher than those of the brachioradialis (*p* < 0.01). The MMT score was positively correlated with the mean and peak RMS values of the paretic biceps and brachioradialis (*r* = 0.89, *r* = 0.91, *r* = 0.82, *r* = 0.85; *p* < 0.001). During FPE exercise, the RMS values (mean and peak) of the biceps and brachioradialis on the paretic side were significantly higher than those on the nonparetic side (*p* < 0.01), and the RMS values (mean and peak) of the brachioradialis on the paretic side were significantly higher than those of the biceps (*p* < 0.01). TheMAS score was positively correlated with the mean RMS of the paretic biceps and brachioradialis (*r* = 0.62, *p* = 0.021; *r* = 0.74, *p* = 0.004), and the MAS score was positively correlated with the peak RMS of the paretic brachioradialis (*r* = 0.59, *p* = 0.029) but had no significant correlation with the peak RMS of the paretic biceps (*r* = 0.49, *p* > 0.05).

**Conclusions:**

The results confirm that the biceps is a vital muscle in active elbow flexion and that the brachioradialis plays an important role in elbow flexor spasticity, suggesting that the biceps should be the focus of muscle strength training of the elbow flexors and that the role of the brachioradialis should not be ignored in the treatment of elbow flexor spasticity. This study also confirmed the application value of sEMG in the objective assessment of individual muscle strength and spasticity in stroke patients.

## 1. Introduction

Stroke is a destructive neurological disease with high morbidity, disability, and mortality. It is the second leading cause of death and the leading cause of disability in the world [1, 2]. Approximately two-thirds of stroke survivors exhibit moderate to severe motor dysfunction, mainly manifesting as muscle weakness, spasticity, and coordination disorder, which greatly affects the quality of life of the patient [3]. Early effective rehabilitation training can greatly promote the functional recovery of patients, and the formulation of precise rehabilitation training programs depends on a detailed rehabilitation assessment. The current clinical rehabilitation assessment of muscle strength mainly involves a manual muscle test (MMT). This method is simple and convenient but also subjective. More importantly, an MMT can evaluate only the overall strength of a group of muscles and is not accurate for a single muscle [4]. Commonly used assessment methods of spasticity are clinical scales, including the modified Ashworth scale (MAS), the clinical spasticity index (CSI), and the Tardieu scale (TS); of these scales, the MAS is the most widely used [5]. These methods are relatively mature in terms of testing procedures, but the results are subjective and difficult to accurately quantify due mainly to the tester's qualitative or semiquantitative evaluation of muscle tension [6, 7]. More importantly, these evaluation methods cannot be subdivided into specific muscles. Taking the elbow flexor muscles as an example, the MAS can only assess the overall muscle tension of the elbow flexor muscles and cannot assess the muscle tension of the biceps and brachioradialis individually. Thus, an important issue to be solved in the clinical rehabilitation of stroke is how to more objectively assess the strength and tension of individual muscles and more precisely analyze the dysfunction of stroke survivors.

Surface electromyography (sEMG) analyzes the bioelectric signals generated during various voluntary and involuntary movements of the neuromuscular system and is performed by guiding, amplifying, recording, and displaying the signals on the skin surface of the detected muscle through surface electrodes. In recent years, as an objective indicator of muscle strength and functional status assessment, sEMG has been widely used in clinical research in the field of neurorehabilitation. For example, Vinstrup et al. measured the sEMG activity of lower limb muscles and confirmed that bodyweight exercises can effectively activate most of the lower limb muscles of stroke patients [8]. Zanin et al. also used sEMG to evaluate the changes in the muscle strength of the wrist flexor muscles before and after acupuncture in stroke patients to verify the treatment effect [9]. In the case of effective control of the interference of measurement factors, the changes in sEMG signals can more objectively reflect the state of muscle function, muscle strength, and coordination of multiple muscle groups. The root mean square (RMS) is the most commonly used and reliable sEMG time-domain analysis index. The RMS value is determined by the change in the sEMG signal amplitude and reflects the change in the muscle contraction intensity. During voluntary exercise, the RMS value reflects changes in muscle strength, while during passive exercise, the RMS value may reflect the degree of muscle tension [10, 11]. Currently, sEMG is used mainly as an objective indicator of muscle strength in many clinical studies. Some studies have tried to adopt sEMG parameters for evaluating muscle spasms after stroke. The cocontraction index (CCI), which was calculated from raw sEMG data, was used to reflect the level of isolated movement across joints and the degree of muscle spasticity [12]. Therefore, one limitation of these previous studies is that they did not directly use sEMG parameters, such as the RMS, to measure spasms in individual muscles in stroke patients.

Previous studies have shown that sEMG can reflect the recruitment of motor units during muscle contraction by estimating the conduction velocity of muscle fibers, thereby objectively evaluating muscle strength [13, 14]. Muscle strength refers to the force of active muscle contraction, and muscle tension is essentially a kind of stretch reflex, which is the reflexive contraction of muscles in the process of passive stretching. Therefore, we reasonably infer that the changes in muscle contraction intensity during passive stretching detected by sEMG can also reflect muscle tension. Rudroff et al. simultaneously used intramuscular electromyography (EMG) and sEMG to record the EMG signal amplitude of the elbow flexor muscles during a submaximal isometric contraction and measured the muscle architecture through ultrasound [15]. It was found that the amplitude recorded by sEMG of the biceps and brachioradialis increased at a faster rate than that recorded by intramuscular EMG at different depths. This indicates that sEMG is more effective than intramuscular EMG in reflecting the overall function of the detected muscle group. Caufriez et al. analyzed the role of the brachioradialis in elbow flexion by sEMG and found that the electromyographic activity of the brachioradialis was much higher during maximum isometric contraction than during maximal voluntary contraction, especially under forearm supination [16]. The above studies have indicated that sEMG can objectively and quantitatively assess the muscle strength of stroke patients and demonstrate its potential for assessing muscle tone, revealing the important role of the brachioradialis in active elbow flexion. However, none of these studies combined muscle strength and muscle tension to comprehensively analyze the influence of the biceps and brachioradialis on elbow flexor activity.

Previous studies have demonstrated that the RMS value representing the amplitude of the EMG signal is correlated with the stretch reflex threshold, suggesting the application prospects of sEMG in the assessment of spasticity [17]. Pandyan et al. used sEMG and the MAS to quantify the clinical efficacy of botulinum toxin in treating elbow flexor spasticity [18]. The results showed that spasticity measured by elbow flexor (biceps) sEMG activity was significantly reduced compared with that before the injection, but the MAS failed to detect this improvement. This suggests that compared with the MAS assessment, sEMG may have higher sensitivity and accuracy in the assessment of spasticity. However, researchers detected the sEMG activity of only the biceps to evaluate elbow flexor spasticity, but the brachioradialis is also an important component of elbow flexion. The contribution of the brachioradialis in the active movement of elbow flexionsuggests that the brachioradialis may also play an important role in elbow flexor spasticity, which is worthy of further research for verification [16].

In this study, we aimed to compare the sEMG activity of the biceps and brachioradialis during maximum voluntary isometriccontraction (MVIC) and fast passive extension (FPE) in stroke patients, analyze the sEMG characteristics of the important muscles that cause elbow flexor weakness and spasticity, and provide a quantitative estimation method for muscle strength and spasticity in stroke patients. We also analyzed the correlation between elbow flexor sEMG activity measured during MVIC exercises and the MMT evaluation results and the correlation between elbow flexor sEMG activity measured during FPE exercises and the MAS assessment results to explore the potential associations between them.

## 2. Materials and Methods

### 2.1. Study Design and Subjects

This study was conducted at the First Affiliated Hospital of Nanjing Medical University from 01 September 2021 to 20 October 2021. The protocol was approved by the Committee of Institutional Ethics (Institutional Review Board, 2021-SR-448) and registered at the Chinese Clinical Trial Registry (ChiCTR2100051880). Written informed consent was required from all subjects and could be provided by proxies for those unable to sign due to severe motor dysfunction.

The inclusion criteria were as follows: (1) age 18-80 years; (2) ischemic or hemorrhagic stroke confirmed with computed tomography (CT) or magnetic resonance imaging (MRI); (3) first-ever episode of hemispheric stroke and poststroke duration > 2 months; (4) unilateral movement impairment; (5) impairment of upper limb (elbow flexors) function, namely, an MMT score of at least 2 and an MAS score of 2 or 3; (6) normal weight, 18.5 ≤ body mass index (BMI) < 24; (7) able to verbally respond to instructions; and (8) stable vital signs (systolic blood pressure of 90-140 mmHg, heart rate of 50-100/min, body temperature < 37.5°C, and blood oxygen saturation > 95%). Patients were excluded if (1) they had contracture of the elbow joint; (2) their condition was complicated with inflammation, pain, osteoarthrosis, or recent injury of an upper extremity; (3) they had other neurological, hematological system, cardiovascular, metabolic, or chronic hepatorenal diseases, a tumor or were pregnant; (4) they had severe cognitive or mental dysfunction; or (5) they were currently enrolled in another trial or participated in a clinical trial within 6 months.

### 2.2. Experimental Procedure

**Figure 1:**
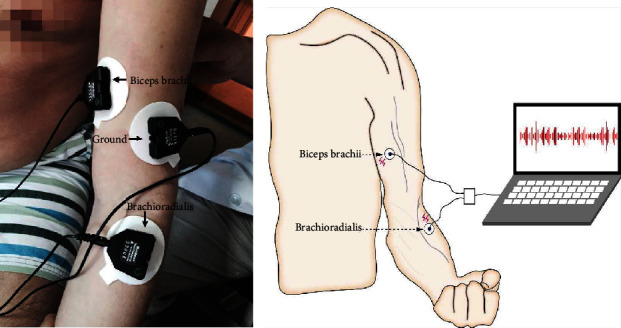
Position of the electrodes on the left upper limb. (a) Electrode locations for the subjects in test. (b) Schematic drawing of electrode positions.

The sEMG signals were sampled using the FlexComp Infiniti system (Model SA7550, Thought Technology, Montreal, QC, Canada, 2048 Hz), which is a 10-channel multimodality encoder for real-time computerized physiological, biofeedback, and data acquisition. Disposable electrodes (Kendall, Medtronic, Minneapolis, MN, USA) were used for all sEMG recordings. Measurement was performed following the recommendations of the SENIAM project (Surface EMG for Non-Invasive Assessment of Muscles) [19]. All tests for all subjects were carried out at the same time of day in the same dedicated room at 22-28°C by a full-time inspector. Before sEMG recording, elbow flexor muscle tension was assessed with the MAS, and muscle strength was assessed with the MMT. Prior to the MMT assessment, subjects underwent 10 minutes of elbow flexor stretching on the paretic side to minimize the impact of elbow flexor spasm on the MMT assessment. BMI values were calculated to ensure that every subject met our inclusion criteria. The central skin surface of the biceps and brachioradialis belly was prepared and cleaned carefully with 75% alcohol. Then, an electrode plate (containing two detection electrodes 2 cm apart) was placed parallel to the muscle fiber orientation, and the electrodes were attached on the skin surface of the test muscle with bandages. The biceps surface electrode was placed on the line between the medial acromion and the fossa cubit 1/3 away from the fossa cubit, while the brachioradialis surface electrode was placed on the lateral side of the forearm 4 cm from the lateral epicondyle. The reference electrode was placed on the lateral epicondyle of the humerus (SENIAM recommendations, Figure 1) [19].

The following group of movements was completed by each subject: (1) MVIC of the nonparetic elbow flexors for 5 seconds, (2) MVIC of the paretic elbow flexors for 5 seconds, (3) FPE of the nonparetic elbow flexors conducted by assessors, and (4) FPE of the paretic elbow flexors conducted by assessors. The subjects rested for 10 minutes between each exercise session to ensure adequate relaxation before moving on to the next group of exercises. Before MVIC exercise, the inspector performed 10-minute stretch training on the paretic elbow flexor of the subject to reduce the influence of muscle spasm on isometric contraction in elbow flexion. During MVIC, each subject was required to sit with the shoulder slightly flexed, forearm supinated, wrist in a neutral position, and elbow placed at 45° of flexion. The inspector fixed the elbow with one hand and applied resistance to the wrist with the other hand. The subjects were instructed to flex their elbow with maximum strength against the resistance of the inspector and sustain the position for 5 seconds. This was repeated three times at an interval of 60 seconds. The maximal values from the three repetitions were taken as the MVIC. During FPE, each subject was instructed to maintain the sitting position with the shoulder and elbow both in a neutral position and forearm supinated, which made it easier to achieve a relaxed state. Our inspectors fixed the elbow with one hand and held the wrist with the other to assist the subject in completing fast passive elbow extension within 1 second. Since a spasm is characterized by a velocity-dependent hypertonic stretch reflex, we set the fast draft time to less than 1 second. This was repeated three times at an interval of 60 seconds. The maximal values from the three repetitions were taken as the FPE measurement. The subjects were allowed to practice in advance to ensure that all the participants could understand and cooperate with the MVIC and FPE measurements.

### 2.3. Data Preprocess

The EMG data were sampled at 2048 Hz. The sEMG signals during MVIC and FPE of the paretic and nonparetic elbow flexors were entered into a computer and analyzed using BioNeuro Infiniti Version 5.0 software (Thought Technology Ltd., Montreal West, QC, Canada). The raw EMG signals were rectified by calculating the RMS value using the following formula:

(1) $$\begin{array}{c} \text{RMS}=\begin{array}{c} t\\ \sqrt{\left({}_{t}^{t+T}\int {EMG}^{2}\left(t\right)dt/T\right)} \end{array}. \end{array}$$

The mean and peak values of the RMS were selected for the analysis. The RMS value of the EMG signals was used to express the muscle activation intensity. During MVIC, a higher RMS value represented the recruitment of more motor units and better muscle strength [9]. We compared the recruitment of motor units between the paretic and nonparetic elbow flexors (biceps and brachioradialis) by analyzing the RMS values during MVIC. Additionally, we analyzed the main muscle groups on the paretic side related to the elbow flexor muscle strength by comparing the RMS values of the biceps and brachioradialis during MVIC. Finally, we analyzed the correlation between the RMS value of the elbow flexors collected during MVIC and the MMT score of the elbow flexor on the paretic side. During FPE, a larger RMS value represented more apparent muscle tension and higher spasticity of the muscle groups [6, 20]. We first compared spasms of the paretic and nonparetic elbow flexors by analyzing the RMS values during FPE. Additionally, we analyzed the contribution of the biceps and brachioradialis to elbow flexor spasticity by comparing the RMS values of the biceps and brachioradialis during FPE. Finally, we analyzed the correlation between the RMS value of the elbow flexors collected during FPE and the MAS score of the elbow flexor on the paretic side.

### 2.4. Statistical Analysis

An equivalence test of mean and standard deviation using one-sided tests on data from a parallel-group design achieved sample size of 14 per group upon 82% power at a 5% significance level when the true difference between the means was 15.00 (estimated based on our pilot results) and the standard deviation of 10.00 (equivalence limits of -25.00 and 25.00). Therefore, we set the final sample size of 15 per group in the current study.

All statistical analyses were performed with SPSS software 25.0 (Chicago, USA). EMG data were analyzed using BioNeuro Infiniti Version 5.0 software. The mean and peak RMS values during MVIC or FPE of the biceps and brachioradialis with the paretic and nonparetic sides were normally distributed and satisfied homogeneity of variance. Continuous variables are presented as the means and standard deviations. Categorical variables are presented as numbers and percentages. The differences in the RMS values of the elbow flexor muscles on the paretic and nonparetic sides and the RMS values of the homolateral biceps and brachioradialiswere compared using a two-tailed paired sample *t* test. The multiclassification MAS and MMT scores were assigned according to order. The MAS had possible scores of 0, 1, 1+, 2, 3, and 4, and a score of 0-5 was assigned. The MMT scores were divided into 6 grades (ranging from 0-5), and a score of 0-5 was assigned. Correlations between the RMS value and the MMT score of the paretic elbow flexor muscles during the MVIC exercise were estimated with the Spearman correlation analysis. Differences between groups were considered significant when *p* values were less than 0.05.

## 3. Results

**Figure 2:**
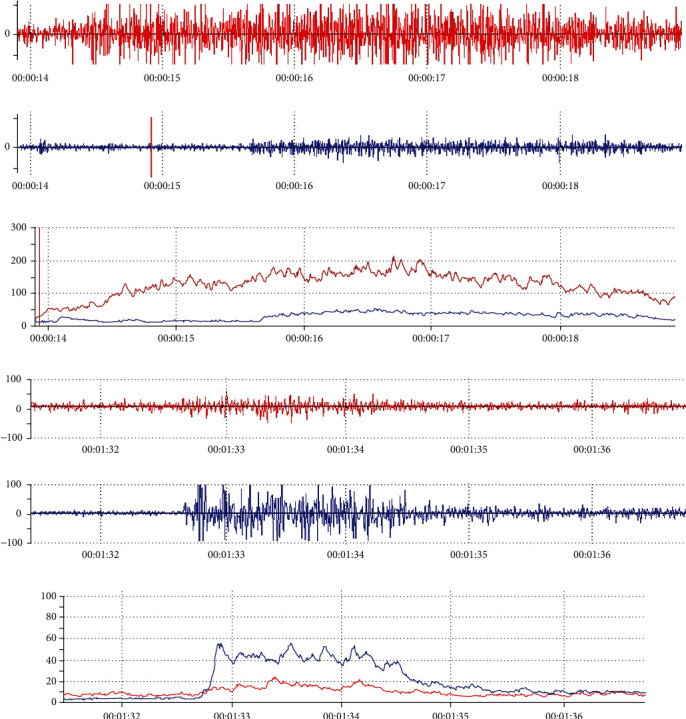
Raw sEMG signals and RMS values of the biceps and brachioradialis during MVIC and FPE on the paretic side. (a) Raw sEMG signals of the biceps brachii and brachioradialis during MVIC. (b) RMS values of the biceps brachii and brachioradialis during MVIC. (c) Raw sEMG signals of the biceps brachii and brachioradialis during FPE. (d) RMS values of the biceps brachii and brachioradialis during FPE. sEMG: surface electromyography. MVIC: maximum voluntary isometriccontraction; FPE: fast passive extension; RMS: root mean square. (red: biceps brachii; blue: brachioradialis).

In this study, we enrolled a total of 15 chronic poststroke hemiplegic patients (aged 50.93 ± 11.98, 10 males and 5 females) with different degrees of elbow flexor spasticity (MAS = 2, 3)from outpatient clinic (Table 1). Nine patients had experienced an ischemic stroke, and six had experienced an intraparenchymal hemorrhagic stroke. The poststroke duration ranged from 2 to 16 months, and the average disease duration was 7 months. There were more subjects with hemiparesis of the left side (*n* = 11, 73.3%). Because subcutaneous fat layer thickening could indeed influence the sEMG signal, the BMI values of the 15 patients were estimated. The results showed that there were no abnormal BMI values and that the mean BMI of the patients was 22.71. All patients completed the study. After processing the raw EMG signals of the biceps and the brachioradialis during MVIC or FPE, the RMS value was computed.  Figure 2 shows the raw EMG signals and RMS values of the paretic biceps and brachioradialis during MVIC and FPE from one subject selected randomly from the 15 subjects.

### 3.1. sEMG Comparative Analysis

**Figure 3:**
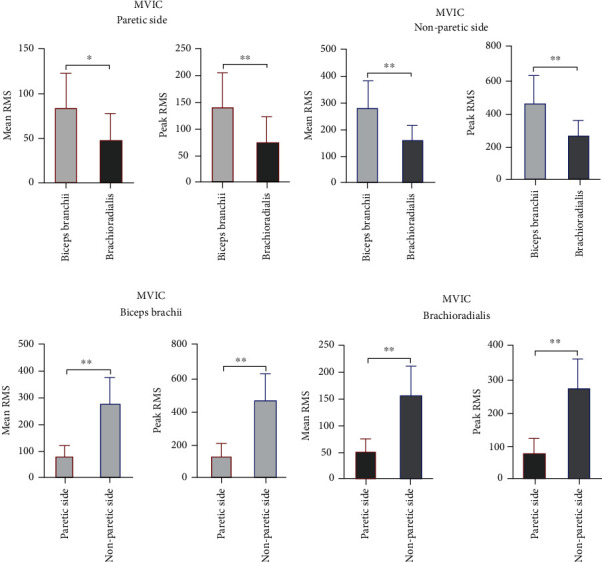
sEMG signals of the elbow flexor muscles (the biceps brachii and brachioradialis) during MVIC. (a) Mean RMS values of the biceps brachii and brachioradialis during MVIC on the paretic side. (b) Peak RMS values of the biceps brachii and brachioradialis during MVIC on the nonparetic side. (c) Mean RMS values of the biceps brachii during MVICon the paretic and nonparetic sides. (d) Mean RMS values of the biceps brachii during MVIC on the paretic and nonparetic sides. sEMG: surface electromyography; MVIC: maximum voluntary isometric contraction; RMS: root mean square; ^∗^*p* < 0.05; ^∗∗^*p* < 0.005.

To analyze the effects of the biceps and brachioradialis during MVIC, the sEMG signals of the elbow flexor muscles (the biceps and brachioradialis) on the paretic side were compared with those on the nonparetic side. To distinguish between the biceps and the brachioradialis, the sEMG signals of those muscles were also compared. The results showed that the RMS values during MVIC of the biceps (mean RMS: 84.70 ± 37.93; peak RMS: 141.65 ± 65.69) and brachioradialis (mean RMS: 48.82 ± 28.33; peak RMS: 77.92 ± 43.34) were significantly lower on the paretic side than on the nonparetic side (*p* < 0.001). Meanwhile, compared with those of the brachioradialis, the mean and peak RMS values of the biceps were significantly higher (*p* = 0.007) (Figure 3).

**Figure 4:**
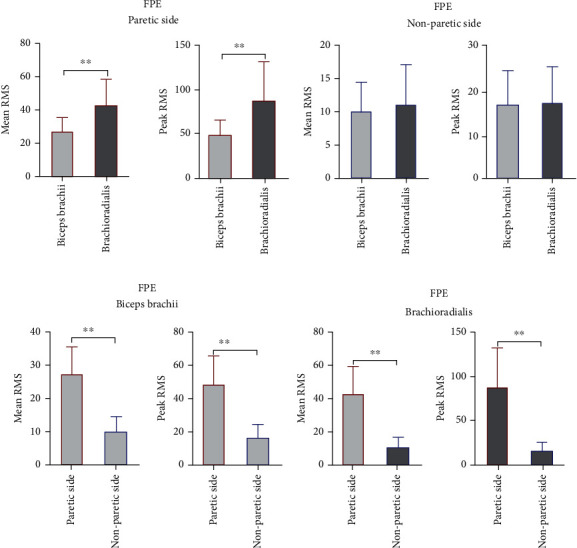
sEMG signals of the elbow flexor muscles (the biceps brachii and brachioradialis) during FPE. (a) Mean RMS values of the biceps brachii and brachioradialis during FPE on the paretic side. (b) Peak RMS values of the biceps brachii and brachioradialis during FPE on the nonparetic side. (c) Mean RMS values of the biceps brachii during FPE on the paretic and nonparetic sides. (d) Mean RMS values of the biceps brachii during FPEon the paretic and nonparetic sides. sEMG: surface electromyography; FPE: fast passive extension; RMS: root mean square; ^∗^*p* < 0.05; ^∗∗^*p* < 0.005.

To gain insight into the role of the biceps and brachioradialis in elbow flexor spasticity, the sEMG signals of the elbow flexor muscles (the biceps and brachioradialis) on the paretic side during FPE were compared with those on the nonparetic side. To further characterize the effect of the biceps and brachioradialis, a comparison of the sEMG signals of those two muscles was performed. The results showed that the RMS values during FPE of the biceps (mean RMS: 27.01 ± 8.57; peak RMS: 48.79 ± 15.35) and brachioradialis (mean RMS: 42.96 ± 16.06; peak RMS: 86.82 ± 43.02) on the paretic side were significantly higher than those on the nonparetic side (*p* < 0.001). Moreover, compared with those of the biceps on the paretic side, the mean and peak RMS values of the brachioradialis were significantly higher (*p* = 0.003) (Figure 4).

### 3.2. Correlation between sEMG Values and Elbow Flexor Muscle Strength

**Figure 5:**
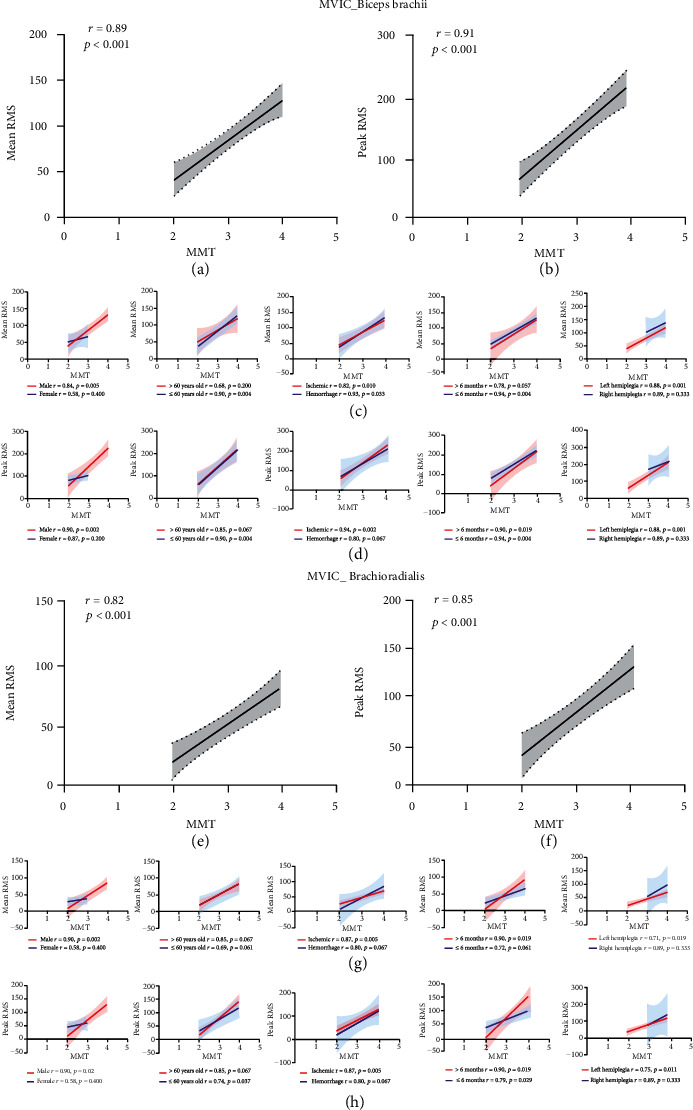
Correlation between sEMG values (mean and peak RMS values) and elbow flexion (biceps brachii and brachioradialis) muscle strength during MVIC on the paretic side. (a) Correlation between MMT score and mean RMS values of the biceps brachii during MVIC. (b) Correlation between MMT score and peak RMS values of the biceps brachii during MVIC. (c) Correlation between MMT score and mean RMS values of the biceps brachii during MVIC in subgroups (sex, age, stroke type, poststroke duration, and hemiparetic side (left/right)). (d) Correlation between MMT score and peak RMS values of the biceps brachii during MVIC in the subgroups (sex, age, stroke type, poststroke duration, and hemiparetic side (left/right)). (e) Correlation between MMT score and mean RMS values of the brachioradialis during MVIC. (f) Correlation between MMT score and peak RMS values of the brachioradialis during MVIC. (g) Correlation between MMT score and mean RMS values of the brachioradialis during MVIC in subgroups (sex, age, stroke type, poststroke duration, and hemiparetic side (left/right)). (h) Correlation between MMT score and peak RMS values of the brachioradialis during MVIC in the subgroups (sex, age, stroke type, poststroke duration, and hemiparetic side (left/right)). sEMG: surface electromyography; MVIC: maximum voluntary isometric contraction; MMT: manual muscle test; RMS: root mean square.

The correlations between the mean and peak RMS values of the paretic biceps and brachioradialis during MVIC and the MMT score and the elbow flexor muscle strength are shown in Figures 5(a), 5(b), 5(e), and 5(f). Statistically significant correlations were detected between the mean RMS value of the paretic biceps during MVIC and elbow flexor muscle strength (*r* = 0.89, *p* < 0.001), the peak RMS value of the paretic biceps muscle during MVIC and elbow flexor muscle strength (*r* = 0.91, *p* < 0.001), the mean RMS value of the paretic brachioradialis during MVIC and elbow flexor muscle strength (*r* = 0.82, *p* < 0.001), and the peak RMS value of the paretic brachioradialis during MVIC and elbow flexor muscle strength (*r* = 0.85, *p* < 0.001).

Five prespecified subgroup analyses (sex, age, stroke type, poststroke duration, and hemiparetic side (left/right)) were carried out. The results showed that the mean RMS value of the paretic biceps muscle during MVIC was associated with elbow flexor muscle strength in males, those aged no more than 60 years, those with a poststroke duration of less than 6 months, and those with hemiparesis on the left side (Figure 5(c)). Meanwhile, the peak RMS value of the paretic biceps muscle during MVIC was associated with elbow flexor muscle strength in male patients, those aged no more than 60 years, those with ischemic stroke, and those with a poststroke duration of less than 6 months (Figure 5(d)). Moreover, the mean RMS value of the paretic brachioradialis during MVIC was associated with elbow flexor muscle strength in male patients, those with ischemic stroke, those with a poststroke duration of more than 6 months, and those with hemiparesis on the left side (Figure 5(g)). Finally, the peak RMS value of the paretic brachioradialis during MVIC was associated with elbow flexor muscle strength in male patients, those aged less than 60 years, those with ischemic stroke, and those with hemiparesis on the left side (Figure 5(h)).

### 3.3. Correlation between sEMG Values and Elbow Flexor Muscle Tension

**Figure 6:**
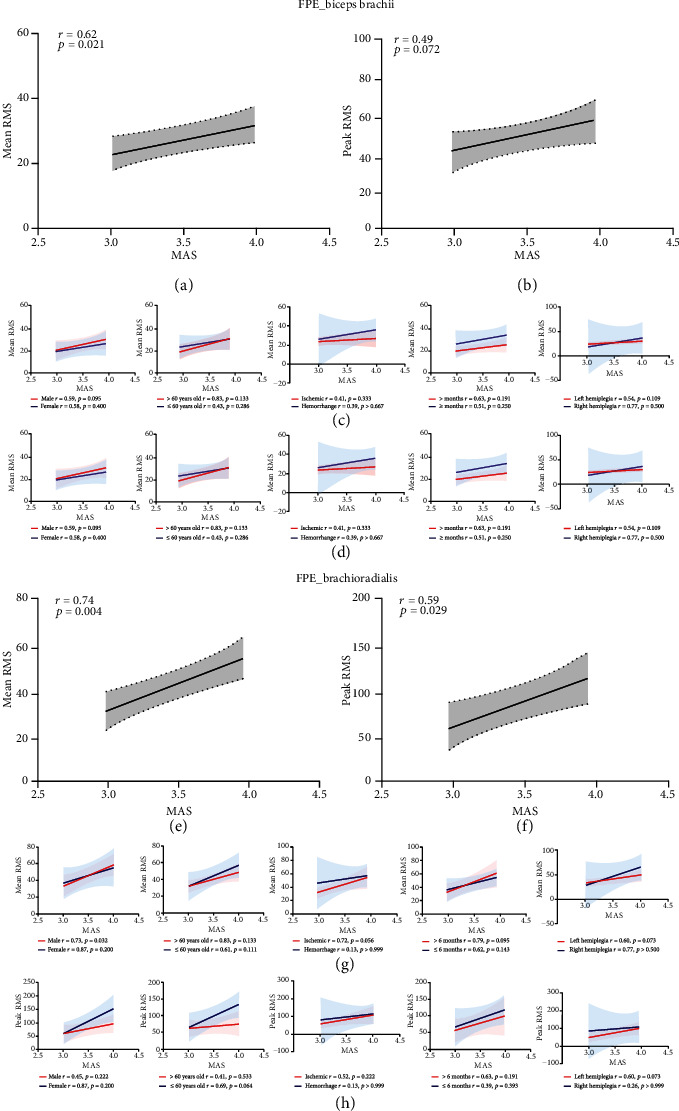
Correlation between sEMG values (mean and peak RMS values) and elbow flexion (biceps brachii and brachioradialis) muscle tension during FPE on the paretic side. (a) Correlation between MAS score and mean RMS values of the biceps brachii during FPE. (b) Correlation between MAS score and peak RMS values of the biceps brachii during FPE. (c) Correlation between MAS score and mean RMS values of the biceps brachii during FPE in the subgroups (sex, age, stroke type, poststroke duration, and hemiparetic side (left/right)). (d) Correlation between MAS score and peak RMS values of the affected biceps brachii during FPE in the subgroups (sex, age, stroke type, poststroke duration, and hemiparetic side (left/right)). (e) Correlation between MAS score and mean RMS values of the brachioradialis during FPE. (f) Correlation between MAS score and peak RMS values of the brachioradialis during FPE. (g) Correlation between MAS score and mean RMS values of the brachioradialis during FPE in the subgroups (sex, age, stroke type, poststroke duration, and hemiparetic side (left/right)). (h)Correlation between MAS score and peak RMS values of the brachioradialis during FPE in the subgroups (sex, age, stroke type, poststroke duration, and hemiparetic side (left/right)). sEMG: electromyography; FPE: fast passive extension; MAS: modified Ashworth scale; RMS: root mean square.

Correlation analysis of the mean and peak RMS values of the paretic biceps and brachioradialis during FPE and the MAS score for muscle tension in elbow flexion was also conducted. Statistically significant correlations were detected between the mean RMS values of the paretic biceps muscles during FPE and muscle tension in elbow flexion (*r* = 0.62, *p* = 0.021), the mean RMS values of the paretic brachioradialis during FPE and muscle tension in elbow flexion (*r* = 0.74, *p* = 0.004), and the peak RMS value of the paretic brachioradialis during FPE and muscle tension in elbow flexion (*r* = 0.59, *p* = 0.029) (Figures 6(a), 6(e), and 6(f)). However, there was no significant association between the peak RMS value of the paretic biceps muscles during FPE and muscle tension in elbow flexion (*r* = 0.49, *p* = 0.072) (Figure 6(b)).

Five prespecified subgroup analyses (sex, age, stroke type, poststroke duration, and hemiparetic side (left/right)) were also performed. The results showed one significant interaction in the prespecified subgroup analysis by sex, whereby the mean RMS value of the paretic brachioradialis during FPE was associated with muscle tension in elbow flexion in men (Figure 6(g)). However, there were no statistically significant differences among the subgroups with respect to the mean or peak RMS value of the paretic biceps muscles during FPE, the MAS score for muscle tension in elbow flexion, the peak RMS value of the paretic brachioradialis during FPE, or the MAS score for muscle tension in elbow flexion (Figures 6(c), 6(d), and 6(h)).

## 4. Discussion

Stroke often leads to severe impairment of limb function and is associated with a decreased quality of life. Muscle weakness and spasm are the main factors affecting the functional recovery of stroke patients. To formulate an optimized rehabilitation program to maximize the functional recovery of patients, a reliable rehabilitation assessment is essential.

Currently, we often use the MMT for muscle strength assessment and the MAS for spasticity assessment in our clinical work. Both of these are semiquantitative assessment methods that are easy to perform but are susceptible to many unstable or uncontrollable factors. More importantly, these clinical scales are for the whole muscle group and cannot measure individual muscles. In addition, some detailed characteristics of the data are lost in the statistical processing of ordered multiclassification data, such as the MMT and MAS data, in clinical studies. In contrast, the continuous variable of sEMG can more comprehensively reflect the actual functional level of patients. Therefore, the objective and quantitative estimation method of sEMG was selected to compare the muscle strength and muscle tension levels of the biceps brachioradialis and brachioradialis in stroke patients, analyze the main muscle groups that affect the muscle strength and muscle tension of elbow flexor muscles, and explore a more objective evaluation to guide the optimization of clinical rehabilitation treatment programs.

sEMG is an objective, noninvasive method for neuromuscular function examination that has the advantages of real-time multitarget measurement and repeatability. It is used for quantitative and qualitative analyses of neuromuscular activity under various exercise states. Its reliability and validity in muscle strength assessment have been demonstrated in previous studies [21, 22]. sEMG mainly includes data in the time and frequency domains. The time-domain analysis is more mature and reliable and is widely used in clinical research [23]. The RMS value is the most important time-domain analysis index, which describes the effective value of discharge within a period of time, represents change in the myoelectric amplitude, and reflects the number of activated motor units and the degree of synchronization of the motor units involved in the activity. The mean and peak RMS values represent the mean and peak EMG activity during exercise, respectively. The combination of the two can objectively and reliably reflect the detected muscle contraction [24].

To analyze the effect of the biceps and brachioradialis on active contraction and to explore the application value of sEMG in the objective evaluation of muscle force, we first compared the sEMG value of the elbow muscle on the paretic side with that on the nonparetic side during MVIC exercise, and the results showed that the RMS values (mean and peak) of the paretic elbow flexors (biceps and brachioradialis) were significantly lower than those of the corresponding muscles on the nonparetic side (*p* < 0.001). sEMG values mainly reflect the recruitment properties of motor units during active contraction of the detected muscle, and orderly recruitment of motor units is crucial for effective muscle power generation [25, 26]. Hu et al. compared sEMG signals during isometric contraction of the first dorsal interosseous muscle in both paretic and nonparetic muscles of stroke patients and confirmed that stroke can lead to motor unit recruitment modifications in paretic muscles, which is the underlying cause of muscle weakness [27]. Our study also shows that the RMS value of the elbow flexor muscles on the paretic side decreased significantly during MVIC exercise compared with those on the nonparetic side, indicating that the recruitment ability of the paretic limb motor units decreased after stroke and that muscle strength decreased accordingly.

Then, we further compared the sEMG signals of the biceps and brachioradialis and found that the mean and peak RMS values of the paretic biceps were significantly higher than those of the brachioradialis (*p* < 0.001), and the same results were also found for the nonparetic side. The results indicate that the biceps is the muscle group with the higher sEMG signal expression than brachioradialis during active contraction of the elbow flexor muscles on both the paretic and nonparetic sides, suggesting its important role in the strength of the elbow flexor muscles. The biceps has been proven to be the main muscle group in elbow flexion and supination movements by intramuscular EMG [28]. Another study reported that biceps tendon injury can lead to severe impairment of elbow flexion and supination in patients [29]. However, the above studies focused only on the role of the biceps in elbow flexion and did not include the brachioradialis. In our study, multichannel sEMG was used to collect and compare the sEMG signals of the biceps and brachioradialis during the MVIC of the elbow flexor muscle, which further confirmed the vital role of the biceps in the active movement of the elbow flexor muscle through objective quantitative data.

Muscle weakness is a common finding after stroke, and approximately 65% of stroke survivors have obvious muscle paralysis [30]. Functional electrical stimulation is the most commonly used method for improving muscle strength in stroke patients with hemiplegia [31]. Eraifej et al. demonstrated that functional electrical stimulation of the upper extremity within two months after stroke can promote functional recovery and improve self-care in daily life [32]. Functional electrical stimulation with position selection is the key factor influencing the treatment effect. For example, the elbow flexor muscles include the biceps, brachioradialis, and brachialis muscles. There is no objective and detailed rehabilitation evaluation method to provide a reliable answer to the specific choice of muscle as the target of electrical stimulation. In clinical practice, the evaluation method is based mainly on the personal experience of therapists. Our results reveal the vital role of the biceps in the active movement of elbow flexion, indicating that the biceps should be given priority in the selection of electrical stimulation sites for elbow flexion. Whether electrical stimulation of the biceps is more conducive to the improvement of elbow flexor muscle strength than electrical stimulation of the brachioradialis needs further research for verification. Biofeedback and progressive resistance strength training have also proven to be effective treatments for improving the muscle strength of stroke patients [33, 34]. Similar to functional electrical stimulation, the selection of stimulation sites is also an important factor affecting the effect of electronic biofeedback therapy. Combined with the results of this study, we should consider the biceps as the main stimulation target. Our research confirms the important role of the biceps in the maximum isometric contraction of elbow flexion, suggesting that progressive resistance strength training with an emphasis on the biceps by adjusting the training position may be more beneficial to the recovery of muscle strength, and it is worthy of further exploration in clinical research. In addition, the important role of the biceps in the active movement of elbow flexor muscles should also be fully considered when botulinum toxin injection is used to treat elbow flexor spasticity, and the dose of injection should be properly controlled to avoid the potential risk of muscle strength decline caused by botulinum toxin injection, resulting in further aggravation of elbow flexor muscle weakness [35].

Finally, we analyzed the correlation between the RMS value of the elbow flexors collected during MVIC exercise and the MMT score of the elbow flexor muscle strength on the paretic side in stroke patients. The results show that the RMS value of the paretic elbow flexor muscles during MVIC exercise is positively correlated with the MMT score, and this positive correlation was also present in most subgroup analyses. Onishi et al. revealed a quasilinear correlation between the EMG signal and the isometric force of the vastus lateralis [36]. However, the study only observed the vastus lateralis and did not assess other knee extension muscles. Our study explored the correlation between the sEMG signal of elbow flexor muscles (biceps and brachioradialis) and the MMT score, and the results indicated a positive correlation between the sEMG signal and muscle strength, suggesting that sEMG can well reflect the force of elbow flexor muscles.

To analyze the role of the biceps and brachioradialis in elbow flexor spasticity and to explore the application prospect of sEMG in the objective evaluation of spasticity, we first compared the sEMG signals of the elbow flexors on the paretic side with those on the nonparetic side during FPE exercise in stroke patients. The results showed that the RMS values of the biceps and brachioradialis on the paretic side were significantly higher than those of the corresponding muscle on the nonparetic side (*p* < 0.001). Spasticity is characterized by the high excitability of the stretch reflex induced by rapid drafting, which is the main feature of spastic muscle that distinguishes it from normal muscle. Thus, the most direct method for the quantitative assessment of spasticity is to identify the parameters related to the stretch reflex. The literature has confirmed that sEMG signals can effectively distinguish the occurrence of stretch reflexes, thus providing a method for evaluating spasticity [37]. Our study showed a significant difference in the expression of sEMG signals of the biceps and brachioradialis between the paretic and nonparetic sides during FPE exercise, suggesting that both the biceps and brachioradialis are important muscle groups causing elbow flexor spasticity.

Next, we further compared the sEMG signals of the paretic biceps and brachioradialis during FPE exercise. The results revealed that the brachioradialis showed significant reflex contraction during FPE and that both the peak and mean amplitudes of the sEMG signal exceeded those of the biceps. This suggests that the brachioradialis plays a significant role in elbow flexor spasticity in stroke patients, perhaps even exceeding the importance of the biceps, which is generally considered to be the dominant muscle group causing elbow flexor spasticity.

The main purpose of our comparison of the contributions of the biceps and brachioradialis in elbow flexor spasticity was to provide a reference for the treatment of elbow flexor spasticity after stroke. The elbow flexor is an important muscle group in the functional activities of the upper limb, and it is also the site with a high incidence of spasticity. Spasms limit the movement ability of the limb and inhibit the potential for recovery [38]. A total of 19-42.6% of stroke patients has spasticity that requires treatment; among them, up to 97% of patients with poststroke spasticity have moderate to severe motor dysfunction [39, 40]. The treatment of spasticity in the clinic mainly includes botulinum toxin injection, stretch training, posture placement, and range of motion training. Numerous reports have indicated that early local botulinum toxin injections combined with therapeutic training can reduce spasms and improve limb function [41–43]. Objective evaluations of spasms and injection plans (injection site and injection volume) are key factors affecting the efficacy of botulinum toxin injection [44]. In previous studies, botulinum toxin treatment strategies for elbow flexor spasticity all targeted the biceps muscle, and the role of the brachioradialis was often neglected [45, 46]. The literature also reveals that the biceps muscle is the main site of elbow flexor toxin injection, with an injection frequency 2-3 times higher than that of the brachioradialis [47]. However, our study indicates that the brachioradialis plays a noticeable role in elbow flexor spasticity, suggesting that rational allocation of brachioradialis doses should be considered in the formulation of a botulinum toxin injection regimen. Similarly, the effective implementation of therapeutic training depends on the objective evaluation of the spasm to determine the main muscles causing the spasm to more accurately lengthen the spastic muscle. Our study suggests that in rehabilitation training for elbow flexor spasticity, not only the biceps but also the brachioradialis should be considered. The local drafting position and movement should be used to strengthen the brachioradialis stretch to achieve more comprehensive effective control of elbow flexor spasticity.

Finally, we conducted a correlation analysis between the RMS value of the elbow flexors collected during rapid passive drafting of the paretic side and the MAS score. The results revealed that the mean RMS value of the elbow flexors during FPE exercise was positively correlated with the MAS scores. The peak RMS value of the brachioradialis was positively correlated with the MAS score, while the peak RMS value of the biceps was not significantly correlated with the MAS score. In the subgroup analysis, the mean RMS value of the paretic brachioradialis during FPE in the male subgroup was positively correlated with the MAS score, while no significant correlations were found in the other subgroups. Xie et al. demonstrated that there was a correlation between the sEMG amplitude in fast passive movement of the biceps and triceps and the MAS scores of flexor and extensor elbow muscles in stroke patients [48]. However, the study did not assess the brachioradialis, another important muscle of the elbow flexor. We included both the biceps and brachioradialis in our study and demonstrated that the RMS values of the two in FPE exercises are correlated with the elbow flexor MAS scores, demonstrating that both the biceps and the brachioradialis may be important muscle groups affecting elbow flexor spasticity. The results also indicate the application value of sEMG in the evaluation of elbow flexor spasticity. The reason why there is no significant correlation between the peak RMS value of the biceps and the MAS score may be related to the statistical deviation caused by the limited sample size in this study. The nonsignificant correlation in the other subgroups may also be related to the sample size. We will expand the sample size in future studies to further observe the correlation between them.

In this study, sEMG was used to quantitatively analyze the muscle strength and spasm of the biceps brachialis and brachioradialis. The results revealed that the biceps was the vital muscle in active elbow flexion and that the brachioradialis plays a significant role in elbow flexor spasticity. However, there are many factors that affect sEMG detection, such as ambient temperature, body position, subcutaneous fat thickness, and active and passive exercise time. To minimize the interfering factors, this study was designed to adopt a unified approach in terms of the environment, inspector, position (forearm supination) and other factors, including a BMI of 18.5-24.0 according to the inclusion criteria. During the exercise of the MIVC or FPE, each subject rotated the forearm externally fixing the elbow joint on the iliac crest, which was useful to reduce the influence of movement on the detection results. More than those, electrodes were secured with adhesive tape and with a tensor bandage to prevent electrode movement during the experiment. The MVIC time was uniformly set to 5 seconds, and the fast passive extension time was set to be completed within 1 second. To ensure that the patients could achieve maximum muscle contraction, maintain the MVIC for 5 seconds, and fully relax their muscles without resistance in FPE exercise, we trained the patients ten times before the formal test. Each test action was repeated three times, and the maximum value was selected for analysis. Due to the strict limitations of the inclusion criteria, the sample size included in this study was limited. Therefore, the conclusions of this study should be interpreted with caution when applied in clinical practice since they were extracted from a limited sample. Future studies should comprehensively explore the underlying mechanisms using larger sample sizes. The brachialis was not included in this study, mainly because it is located in the deep layer of the lower half of the biceps, and its sEMG signal is covered by the biceps. In addition, only elbow flexor muscle groups were observed in this study, and other paralyzed muscles in stroke patients were not included. Future studies should be extended to other spastic muscles to better elucidate the specific muscles that affect spasticity and provide more comprehensive reference information for the optimization of rehabilitation training programs in clinical practice (the selection of electrical stimulation sites in hemiplegic limbs, the formulation of botulinum toxin injection programs, and the allocation of rehabilitation training priorities), achieve more accurate and efficient rehabilitation treatment, and promote the recovery of patients.

## 5. Conclusions

In this study, sEMG was used to confirm that the biceps plays a vital role in the MVIC of elbow flexion and that the brachioradialis plays a significant role in elbow flexor spasticity. The findings provide a quantitative and objective basis for the optimization of rehabilitation strategies for patients with stroke, such as the selection of electrical stimulation sites for elbow flexion, the focus of muscle strength training, and the administration of botulinum toxin injections. This research should be extended to the various spastic muscle groups of stroke patients, and sEMG should be used to analyze the main muscle groups that affect muscle strength and muscle tension to provide more objective evidence for the formulation of precise rehabilitation training programs.

## Figures and Tables

**Table 1:** Subject demographic information.

Patient No.	Age (years)	Gender	Duration of stroke (months)	Side of hemiparesis (left/right)	Type of stroke	BMI	MMT	MAS
1	40	Female	6	Left	Ischemic	23.24	2	2
2	61	Male	3	Right	Hemorrhagic	23.67	3	3
3	43	Male	10	Left	Hemorrhagic	23.26	3	3
4	56	Female	15	Left	Ischemic	21.37	3	2
5	42	Female	6	Left	Hemorrhagic	22.64	2	3
6	65	Male	6	Left	Ischemic	23.18	3	3
7	39	Male	3	Left	Ischemic	23.81	4	2
8	29	Male	2	Left	Hemorrhagic	23.78	3	2
9	62	Female	8	Left	Ischemic	20.40	3	2
10	63	Male	11	Left	Ischemic	22.94	4	2
11	35	Female	3	Left	Ischemic	23.51	2	3
12	61	Male	8	Left	Ischemic	20.72	2	2
13	53	Male	16	Right	Hemorrhagic	20.98	4	3
14	64	Male	7	Right	Ischemic	23.38	3	2
15	51	Male	4	Right	Hemorrhagic	23.78	4	3

BMI: body mass index; MMT: manual muscle test; MAS: modified Ashworth scale.

## Data Availability

The data used to support the findings of this study are available from the corresponding author upon request.
